# Calibration Analysis of High-G MEMS Accelerometer Sensor Based on Wavelet and Wavelet Packet Denoising

**DOI:** 10.3390/s21041231

**Published:** 2021-02-09

**Authors:** Yunbo Shi, Juanjuan Zhang, Jingjing Jiao, Rui Zhao, Huiliang Cao

**Affiliations:** Science and Technology on Electronic Test & Measurement Laboratory, North University of China, Taiyuan 030051, China; shiyunbo@nuc.edu.cn (Y.S.); s1906105@st.nuc.edu.cn (J.Z.); s1706152@st.nuc.edu.cn (J.J.); zhaorui@nuc.edu.cn (R.Z.)

**Keywords:** MEMS accelerometer, noise reduction, wavelet packet denoising, wavelet denoising, high-G calibration

## Abstract

High-G accelerometers are mainly used for motion measurement in some special fields, such as projectile penetration and aerospace equipment. This paper mainly explores the wavelet threshold denoising and wavelet packet threshold denoising in wavelet analysis, which is more suitable for high-G piezoresistive accelerometers. In this paper, adaptive decomposition and Shannon entropy criterion are used to find the optimal decomposition layer and optimal tree. Both methods use the Stein unbiased likelihood estimation method for soft threshold denoising. Through numerical simulation and Machete hammer test, the wavelet threshold denoising is more suitable for the dynamic calibration of a high-G accelerometer. The wavelet packet threshold denoising is more suitable for the parameter extraction of the oscillation phase.

## 1. Introduction

As the manufacturing process of micromechanical systems (MEMS) continues to evolve, machining accuracy continues to increase. Silicon micromachined accelerometers are widely used in consumer electronics, aerospace, inertial navigation, etc., due to their low manufacturing cost, light weight, and low power consumption [1,2,3,4,5]. Some of these high-G accelerometers can accept up to 10^6^ m/s^2^ acceleration for seismic measurements, collision safety testing, equipment related to nuclear power generation, marine, aerospace equipment, micromotion devices, etc. [3]. High-G accelerometers provide strong support for military and test in special environments. However, MEMS accelerometers often have large errors as part of the vibration measurement system, which reduces their practicability, the most significant of which is the relatively high level of self-noise observed in the output signal [2,6]. Noise limits the measurement resolution, so these sensors cannot be used for accurate diagnostic measurements [7,8]. At the same time, due to the small output of the high-G acceleration sensor, the noise also has a certain impact on its performance. This results in high range acceleration sensors that are often unsatisfactory in terms of repeatability and consistency.

The output signal of a MEMS is a combination of a signal and a variety of noises. The source of noise is mainly composed of low-frequency (long-term) components and high-frequency (short-term) components [9]. The high-frequency component has white noise characteristics, and the low-frequency component has correlated noise characteristics, and the error gradually changes during operation [10]. Traditional denoising generally uses Fourier transform or Kalman filtering. The Fourier transform has low resolution in time domain applications and is not suitable for the processing of narrow pulse width and high impulse signals. The matrix operation used in the denoising of a Kalman Filter makes the calculation time longer and the waveform distortion more serious [11]. Mohammed El-Diasty used Allan analysis of variance and least squares analysis (LSSA) to study the effect of temperature point changes on the MEMS inertial sensor noise model [12]. Some scholars use the Markov random model to denoise graphics or signals [13,14]. For example, Rongqiao Zhang proposes to use a Markov random model to process Bayesian images [13]. Zapata tested the validity of the Gauss–Markov model for bias parameters. The statistical analysis conducted in the work is a key in providing mechanisms for capturing the drift in the fixed pattern noise parameters [14]. Yanyan Li proposed a new data-driven adaptive multi-scale denoising method with the Markov model as the best noise model. The power law and white noise are combined to filter, and the obtained model is the optimal neural network noise model in the study area [15]. In recent years, some scholars have used neural network methods to analyze MEMS devices [16]. However, the Gauss–Markov model does not work well in MEMS cells due to the high sensor inherent errors. On the other hand, the use of neural networks (NN) to model random drift takes a long time, affecting its real-time implementation [5].

In order to overcome the traditional denoising time, the parameter selection is difficult, and the waveform distortion is large, and so on. In view of the application characteristics of MEMS devices, many scholars have proposed a noise reduction method based on wavelet transform. Wavelet transform has the ability to characterize local features in both time and frequency domains. It is more conducive to testing transient and abrupt signals in high G-value environments, compared with traditional methods. Common wavelet denoising methods include modulus maxima denoising method, threshold denoising, and translation invariant method. Derek Abbott solved the problem of extracting noise from electronic stethoscope signals using wavelet and averaging [17]. Xiaobin et al. proposed a threshold denoising method based on wavelet analysis, which can obtain the optimal estimation value in Besov space [18]. Dake Chen proposed a new method for temperature-independent sensitivity optimization of MEMS accelerometers using wavelet neural network [19]. ZeYu Yan proposed a hybrid algorithm that combines Time-frequency peak filtering (TFPF), Local mean decomposition (LMD) and Sample entropy (SE) to reduce noise of high-G MEMS accelerometer signals [20]. For the temperature compensation of high-g MEMS accelerometer (HGMA), Min Zhu proposed four algorithms: radial basis function neural network (RBF NN), RBF NN based on genetic algorithm (GA), RBF NN based on GA with Kalman filter (KF), and the RBF NN + GA + KF method compensated by the temperature drift model [21].

Signal denoising based on wavelet analysis can be realized by wavelet decomposition or wavelet packet decomposition. However, for MEMS high-G accelerometer used in narrow pulse width and high impact environments, the wavelet denoising method should not only remove noise, but also not affect the normal signal analysis. It is indeed a problem. In this paper, for the specific application environment of the MEMS high-G accelerometer, signal denoising is performed by using the wavelet threshold denoising method. Finally, a series of parameters, such as wavelet threshold denoising and signal-to-noise ratio of wavelet packet threshold denoising, are compared to analyze the wavelet denoising method which is more suitable for a high-G accelerometer.

## 2. Algorithm

The wavelet threshold denoising method can also be called the threshold function method. Using the multi-scale characteristics of wavelet analysis, the signal is decomposed by wavelet. Different threshold functions are used to determine whether the wavelet signals of different layers are noise signals. The wavelet coefficients related to noise are directly zeroed (hard threshold method) or become corresponding appropriate values according to different layer numbers (soft threshold method). In this way, the wavelet threshold denoising process is implemented. It can be seen that the selection of the wavelet basis, the number of wavelet decomposition layers, and the threshold value have a great influence on the denoising effect of wavelet threshold denoising. Too many wavelet decomposition layers will cause information loss, signal-to-noise ratio reduction, and increased computational difficulty. If the number of decomposition layers is too small, the noise reduction effect is not obvious [22]. The selection of the threshold directly determines whether the useful signal can be effectively stripped from the original signal. Therefore, this chapter mainly describes the wavelet decomposition layer algorithm and the threshold function algorithm.

### 2.1. Wavelet Adaptive Decomposition

**Figure 1:**
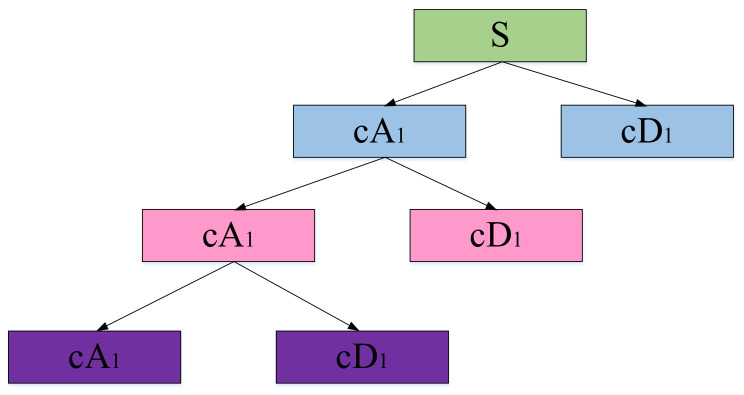
Schematic diagram of wavelet decomposition.

Wavelet decomposition is to decompose the original signal to obtain low-frequency coefficients and high-frequency coefficients. The low-frequency coefficient is decomposed as the input signal of the next stage to obtain a new set of low-frequency coefficients and high-frequency coefficients, and so on, until it is decomposed to the set number of decomposition layers; the maximum number of decomposition layers is log_2_N. The decomposition process is shown in Figure 1.

**Figure 2:**
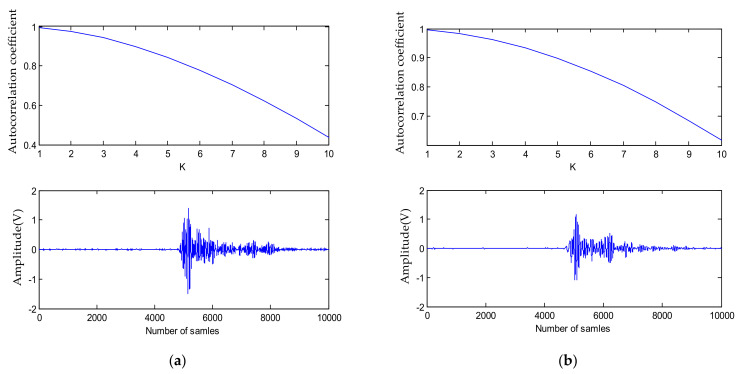
(**a**) Fifth-layer autocorrelation coefficient and detail component; (**b**) sixth-layer autocorrelation coefficient and detail component.

This paper adopts the adaptive decomposition layer number method proposed by Huang [16]. Based on the stationary time series test statistic, the wavelet coefficients of each layer are solved for their autocorrelation coefficients. The autocorrelation function of the discrete time series lag t period is:

(1) $$\rho_{t}=\frac{\sum_{i=1}^{N-t}\left(a_{i}-\bar{a}\right)\left(a_{i+t}-\bar{a}\right)}{\sum_{i=1}^{N-t}{\left(a_{i}-\bar{a}\right)}^{2}}\;t=1,2,\cdots K$$

(2) $$\bar{a}=\frac{1}{N}\sum_{i=1}^{N}a_{i}$$

where *a*_1_, *a*_2_, … *a*_n_ are wavelet coefficients to extract each wavelet layer, *N* is the sampling length, and *K* is generally 5–10. The χ2 hypothesis is used to verify whether the detail coefficient is white noise, layer by layer. When the autocorrelation coefficient of each layer is lower than the verification value, the layer still contains the useful signal. This method is used to verify the number of stratification times in the Machete hammer test. After the hysteresis coefficient is 2, the autocorrelation coefficient is below 0.2, and the sequence is obviously uncorrelated, which is the noise signal. It can be seen from Figure 2 that the details of the sixth layer have already contained useful signals in the wavelet reconstruction coefficients. Therefore, the wavelet decomposition is only decomposed into five layers in the Machete hammer impact test.

### 2.2. Wavelet Packet “Best Tree”

**Figure 3:**
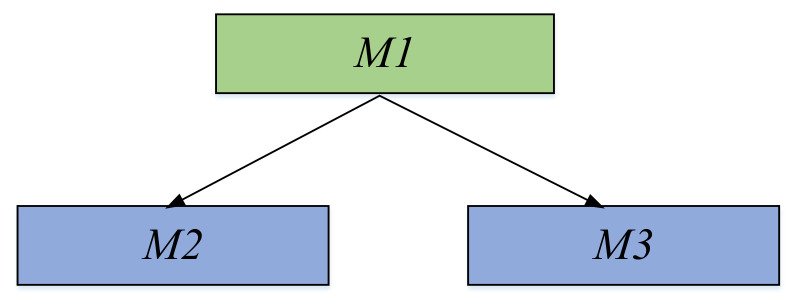
Best tree decomposition.

Different from the wavelet threshold denoising, the wavelet packet is to decompose the original signal to obtain the high-frequency coefficient and the low-frequency coefficient. However, both the high-frequency coefficient and the low-frequency coefficient are used as the input signal of the next stage to be decomposed by analogy, until it is broken down to the set number of layers. The decomposition of the wavelet packet needs to find the “best tree” of the wavelet packet first. This paper uses the Shannon entropy criterion to find the best tree. The Shannon entropy criterion algorithm is as follows:

(3) $$H\left(u\right)=-\sum_{k\in Z}P_{k}logP_{k},P_{k}=\frac{{\left|u\left(k\right)\right|}^{2}}{\sum_{k\in Z}{\left|u\left(k\right)\right|}^{2}}\;\mathrm{or}\;M\left(u\right)=-\sum_{k\in Z}{\left|u_{k}\right|}^{2}log{\left|u_{k}\right|}^{2}$$

where *u_k_* is the wavelet packet coefficient sequence, and the optimal tree of the wavelet packet is determined by calculating the sub-coefficient and the parent coefficient. The selection criterion is referenced in Figure 3. If *M*_1_ > *M*_2_ + *M*_3_, *M*_2_ + *M*_3_ is chosen, otherwise *M*_1_ is chosen. It is calculated by the Shannon entropy criterion that the decomposition of five layers is most suitable in the Machete hammer test environment.

### 2.3. Threshold Function

**Figure 4:**
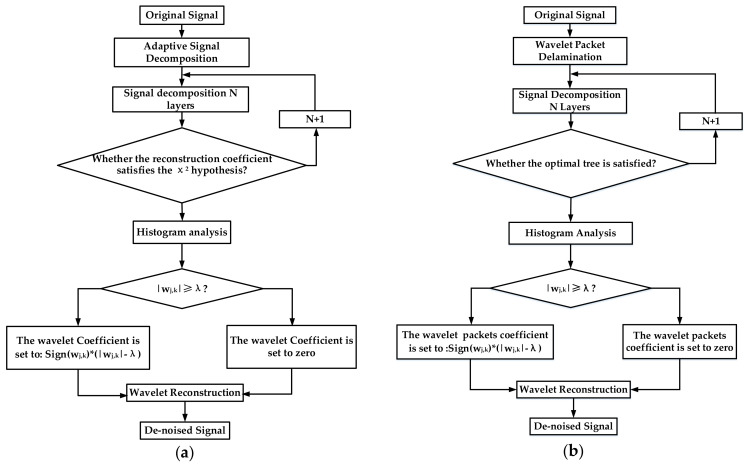
(**a**) Wavelet threshold denoising flow chart; (**b**) wavelet packet threshold denoising flow chart.

Due to the special environment of the high-G acceleration sensor, the impact signal is a high-frequency, high-amplitude signal in the test environment. Therefore, for the target environment, this article uses the Stein unbiased likelihood estimation threshold (SURE) for threshold analysis. Figure 4 shows the wavelet threshold denoising flow chart and wavelet packet threshold denoising flow chart. The unbiased likelihood estimation threshold algorithm is as follows:

The elements in the vector to be estimated are taken to be absolute values, then sorted from smallest to largest, and then each element is squared to get the new vector P (*P*_1_, *P*_2_, … *P_N_*) to be estimated.

Correspondingly to subscript *i* of each element, if the threshold is the square root of the *i*-th element of the vector to be estimated, then the risk algorithm is [11]:

(4) $$r_{i}=\frac{\left[N-2i-(N-i)P_{i}+\sum_{k=1}^{i}P_{k}\right]}{N}\;i=1,2,\cdots ,N$$

According to the obtained risk formular, taking the minimum value *r_a_* in the R element as the risk value, the corresponding threshold is obtained from the subscript vector a of *r_a_*: $th=\sqrt{r_{a}}$. Then, the threshold can be obtained: $\lambda =\sigma \sqrt{P_{a}}$

Since the original signal *x*(*n*) is decomposed by wavelet, the wavelet decomposition detail coefficient $w_{j,k}$ can be obtained, and $w_{j,k}$ is the soft threshold method:

(5) $${\hat{w}}_{j,k}=\left\{\begin{array}{l} sign(w_{j,k})(\left|w_{j,k}\right|-\lambda ),\left|w_{j,k}\right|\geq \lambda \\ 0,\left|w_{j,k}\right|<\lambda \end{array}\right.$$

After the threshold processing, the reconstruction coefficient ${\hat{w}}_{j,k}$ is obtained, and the denoising signal $\hat{x}\left(n\right)$ is obtained from the reconstruction coefficient. The noise standard value σ is expressed as follows, of which median $\left(\left|{\hat{w}}_{j,k}\right|\right)$ is the median of wavelet multi-resolution decomposition coefficient [23,24].

(6) $$\sigma =\frac{median(\left|w_{j,k}\right|)}{0.6745}$$

## 3. High-G MEMS Accelerometer

This paper uses a high-G MEMS accelerometer (HGMA) independently developed by the Science and Technology on Electronic Test & Measurement Laboratory for testing [22].

### 3.1. Structure and Structural Parameters of the HGMA

**Figure 5:**
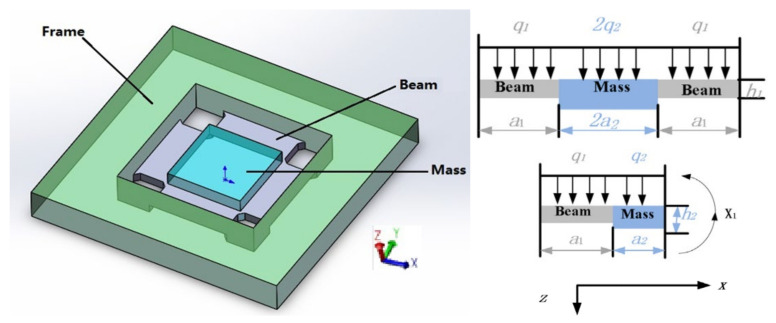
High-G micromechanical systems (MEMS) accelerometer (HGMA) structure schematic and size.

This high-G accelerometer uses a four-cantilever beam structure, as shown in Figure 5. The vibration analysis is based on the theory of Timoshenko beam, and the acceleration acts on the mass to realize the change of the resistance value on the cantilever beam [25].

The high-G accelerometer uses ANSYS software (ANSYS, Inc., South pointe, PA, USA) for modal analysis. The first-order mode is the mass working mode; the second-order mode is the mass moving around the X-axis; the third-order mode is the mass moving around the Y-axis, and the fourth-order is the mass moving around the Z-axis. The specific vibration frequency of each mode is shown in Table 1 [25].

### 3.2. Process of the HGMA

**Figure 6:**
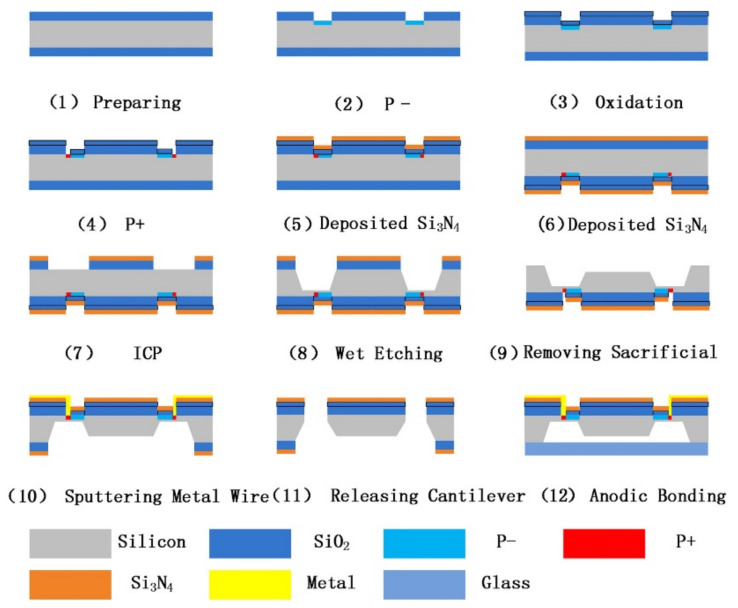
Process of the HGMA.

The main process of the HGMA is shown in Figure 6 [25], including the silicon process flow and the glass process flow. N-type double-sided oxidized silicon wafer (with 350 μm thickness) was selected as the substrate material, as shown in Figure 6(1). Then, in Figure 6(2), P− parts (concentration is 4 × 10^14^ cm^−2^) are generated on the surface of silicon substrate. In Figure 6(3), in order to protect the P- part and act as a mask for another etching process, another 300 nm thickness SiO_2_ is deposited on top of the SiO2 level. In Figure 6(4), the P+ position is etched by front lithography, and the P+ (concentration is 1 × 10^16^ cm^−2^) parts are generated, thus the piezoresistor parts are accomplished. In Figure 6(5,6), 300 nm-thick Si_3_N_4_ layers are formed on both sides of the silicon wafer by sputtering technology. In Figure 6(7), the Si_3_N_4_ and SiO_2_ levels on the back of the silicon wafer are removed, and inductively coupled plasma etch (ICPE) is used to generate the thickness difference between the beam and the mass (120 μm). Then, the mass (with 200 μm thickness) and beams (with 80 μm thickness) are successively formed on the back of the wafer by wet etching technology, as shown in Figure 6(8). Then, the back-side sacrificial levels (Si_3_N_4_ and SiO_2_) in Figure 6(9) are removed, and the back-side structure is completed. The wafer is turned over and the metal Al is deposited by magnetron sputtering on the front side to form metal wires, as shown in Figure 6(10). After that, the structure is released by ICP etching, as shown in Figure 6(11). Finally, the back side of silicon substrate is bonded with the glass substrate by anode bonding technology.

**Figure 7:**
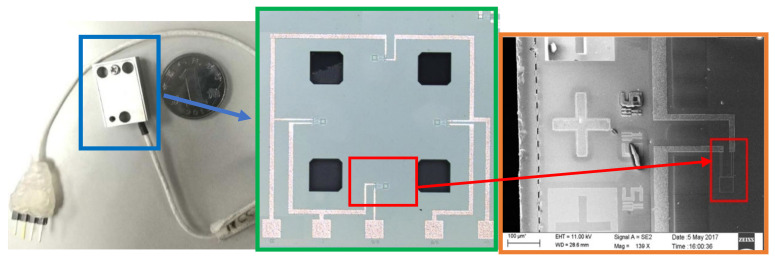
Overall photo, charge coupled device (CCD) photo and SEM photo of the HGMA.

The surface of the chip was magnetron-sputtered and sputtered with aluminum wires. The circuit uses a Wheatstone bridge to convert the acceleration and voltage values through the piezoresistive effect. The design range is 200,000 g, the anti-overload is 250,000 g, and the high-G accelerometer SEM photos and packages are shown in Figure 7 [26].

## 4. Experiment Analysis

### 4.1. Simulation

In order to better compare the two test methods, a shock signal with a pulse width of 30 us was constructed in numerical simulation. In the laboratory test environment of high-G accelerometers, the noise sources are generally circuit noise and white Gaussian noise. Therefore, the signal that is contaminated by noise is set to S.

**Figure 8:**
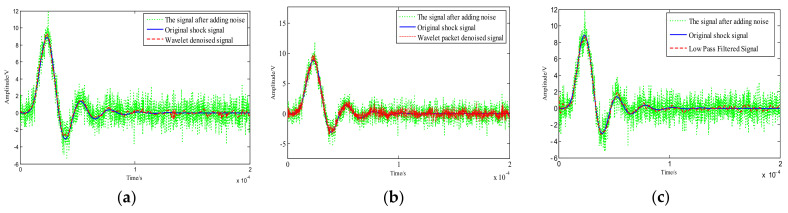
(**a**) Wavelet threshold denoising for the constructed signal; (**b**) wavelet packet threshold denoising for the constructed signal; (**c**) low-pass filtered for the constructed signal.

(7) S=i+i(e)+i(g)

where: *i*(*e*) is collected from the experimental device under static conditions of the sensor, and *i*(*g*) is Gaussian white noise generated from numerical simulation, and the original signal is formed by the superposition of the signals. In order to compare the effect of wavelet decomposition and low-pass filter, this paper used the eighth-order Butterworth low-pass filter for filtering, so as not to attenuate the main peak amplitude, and the filter cutoff frequency was set to 250 KHz. The sym8 wavelet base and wavelet packet were compared by wavelet threshold denoising and wavelet packet threshold denoising. The results are shown in Figure 8.

It can be seen from the waveform before and after denoising that the denoising effect of wavelet threshold denoising and low-pass filtering is obviously better than wavelet packet threshold denoising. At the same time, the waveform reconstructed after the wavelet threshold denoising is smoother and the amplitude is increased to some extent.

In order to quantitatively analyze the denoising ability, the signal-to-noise ratio (SNR) and the root mean square error (RMSE) were taken as the denoising performance indicators. The SNR and RMSE were calculated as follows:

(8) $$SNR=10*\mathrm{lg}\left[\frac{\sum_{n}{\hat{x}}^{2}(n)}{\sum_{n}{\left[\hat{x}(n)-x(n)\right]}^{2}}\right]$$

(9) $$RMSE=\sqrt{\frac{1}{n}\sum_{n}{\left[\hat{x}(n)-x(n)\right]}^{2}}$$

where $\hat{x}(n)$ is the original signal, x(n) is the noise-reduced signal, and *n* is the signal length.

**Figure 9:**
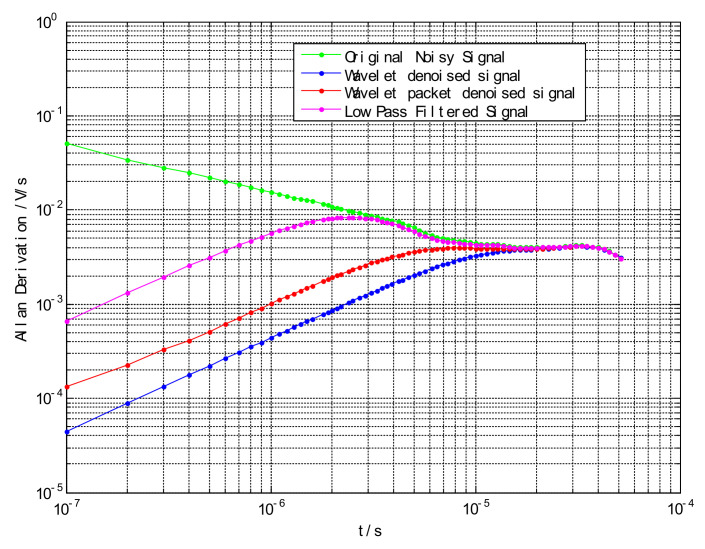
ALLAN deviation map after denoising the airborne sensor signal.

In order to more clearly compare the denoising ability of the three denoising signals, the high-G accelerometer was used to measure the signal in the static condition, and the performance index was evaluated by the ALLAN variance method. The ALLAN variance curve can quantify the equivalent value of acceleration random walk. In Figure 9, the original signal, wavelet threshold denoising, wavelet packet threshold denoising, and low-pass filtering at 10^−7^ are 5.131 × 10^−2^ V/s, 6.68 × 10^−2^ V/s, 1.351 × 10^−4^ V/s, and 4.403 × 10^−5^ V/s.

Through comparison of image (Figure 8 and Figure 9) and denoising performance (Table 2), it can be seen that the signal-to-noise ratio after wavelet threshold denoising is significantly higher than the wavelet packet threshold denoising, and the reconstructed signal has higher smoothness and slightly larger root mean square error.

By comparing the three images and the denoising performance, it can be clearly seen that the wavelet threshold denoising ability using the adaptive algorithm is better in the three denoising algorithms. Its denoising effect is better, the reconstructed signal waveform distortion is small, and the signal-to-noise ratio is high. It can be seen from the ALLAN deviation that wavelet denoising is superior to low-pass filtering in the processing of Machete hammer static calibration.

### 4.2. Experiment and Discussion

#### 4.2.1. Experiment Analysis

**Figure 10:**
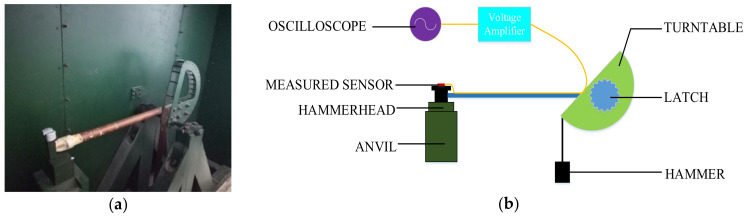
(**a**) Hammer drop device and (**b**) test schematic.

To further verify the denoising ability under the actual impact signal, we tested the sensor using a Machete hammer, as shown in Figure 10. The Endevco voltage amplifier was used to amplify the signal by a factor of 100 and the sensor was supplied with +5 V. The Tektronix MSO 4034B oscilloscope acquires the data and records the test image at a sampling frequency of 10 MHz. The sensor uses cyanoacrylate adhesive to attach the sensor to the Machete hammer, and the impact acceleration is affected by changing the drop height.

The goal of the method is supposed to contain three aspects:

1. Reflect and keep the power in the first sideline cutoff frequency, which means keeping the real peak amplitude at the calibration shock peak frequency.
2. Pick up the frequency information of the resonant frequency of HGMA, and this is helpful to recognize the mechanical characteristic of the HGMA sensor.
3. Cut off the high-frequency noise during the HGMA calibration.

**Figure 11:**
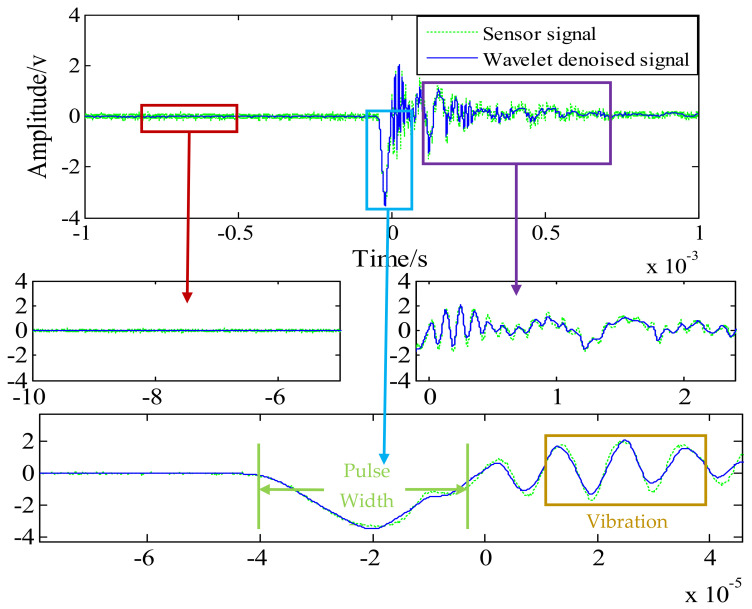
Test signal before and after wavelet threshold denoising.

**Figure 12:**
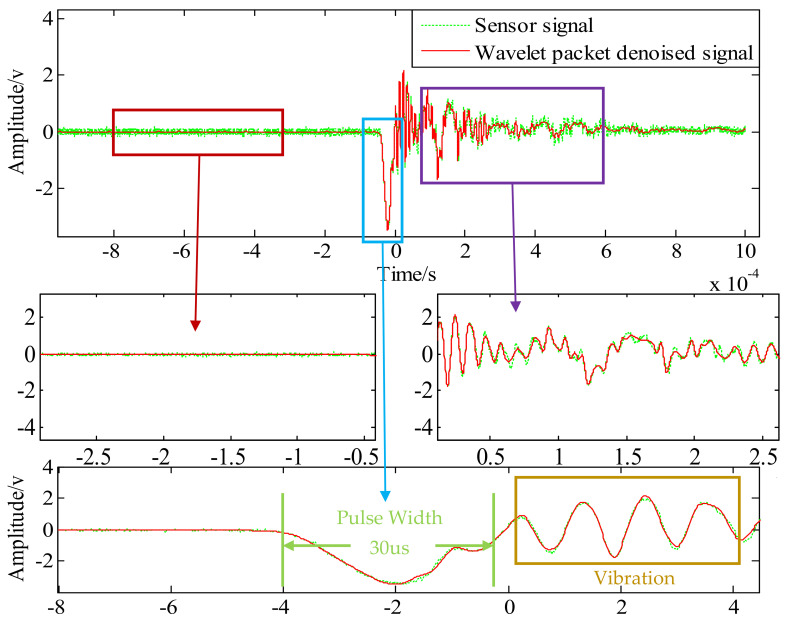
Test signal before and after wavelet packet threshold denoising.

After considering the requirements of continuous symmetry, regularity, and tight support of wavelet base, the ‘dB’ and ‘sym’ wavelet functions were compared, and finally ‘sym8’ was selected as wavelet mother function. It can be concluded from the previous analysis that wavelet denoising decomposes the signal into five layers and six groups of signals, and wavelet packet denoising decomposes the signal into five layers of 32 groups of signals. Both use the Stein unbiased likelihood estimation principle for soft threshold denoising. Figure 11 and Figure 12 show the comparison of signal patterns before and after denoising in two ways.

The original signal data and denoising results of the two methods are shown in Figure 11 and Figure 12. The original signal data is mainly divided into three stages: preparation stage, impact stage, and vibration stage. Due to the problems of the surrounding environment, the amplifier, and the oscilloscope itself, there is a relatively obvious noise signal before the impact, accompanied by the entire test process. It can be seen from Figure 11 and Figure 12 that wavelet threshold denoising and wavelet packet threshold denoising can filter out such signals well. Therefore, the filtering effect of both in the preparation stage is basically the same.

The impact stage is the period of time during which the Machete hammer strikes the first acceleration signal generated by the hammer. The original signal has some distortion due to noise during the impact stage, and a “burr” appears at the peak. Under high impact conditions, the size of the burr will cause an error of 1000–2000 g for the measured acceleration. The peak value of the original signal was about 34,070 g and the pulse width was 30 us. The reconstructed signals of the two wavelet denoising methods do not change the pulse width of the impact signal, and the waveform is smoother. The peak values of the three signals are listed in Table 3. The wavelet threshold denoising signal amplitude is 35,225 g, and the wavelet packet threshold denoising signal amplitude is 34,759 g. By contrast, without changing the pulse width, the wavelet threshold denoising method can increase the amplitude of the signal more than the wavelet packet threshold denoising method.

In the vibration stage, the main cause of the oscillating waveform is the sensor vibration output driven by the overall vibration of the hammerhead and the output signal to the sensor, due to problems such as encapsulation and other packaging. The vibration frequency at this stage is concentrated between 300 KHz and 500 KHz. In this stage, the filtering effect of the wavelet threshold denoising is more obvious after the impact phase. Compared with the wavelet packet threshold denoising, its influence on the oscillation amplitude is obvious.

**Figure 13:**
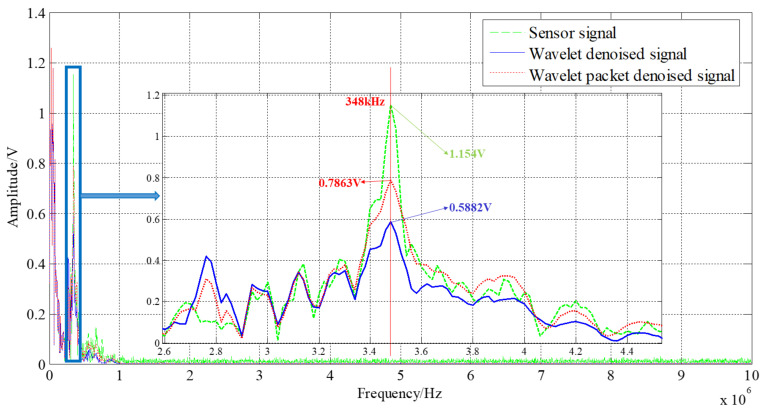
Spectrum before and after denoising.

The spectrum diagram before and after denoising is shown in Figure 13; the “vibration stage” is amplified. The peak frequency of the vibration stage is about 348 KHz, and the amplitude and shape of the wavelet packet threshold denoising results are more in line with the original data. The original signal amplitude is 1.154 V, the wavelet threshold denoising signal amplitude is 0.5882 V, and the amplitude of the wavelet packet threshold denoising signal is 0.7863 V. It shows that the wavelet packet threshold denoising method can better reflect the dynamic characteristics of a high-G accelerometer.

#### 4.2.2. Static Calibration

**Figure 14:**
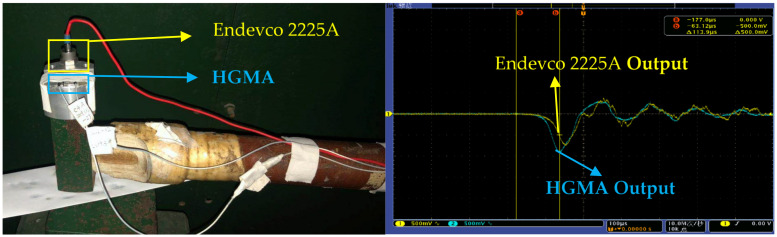
Endevco 2225A reference and calibration result curves.

**Figure 15:**
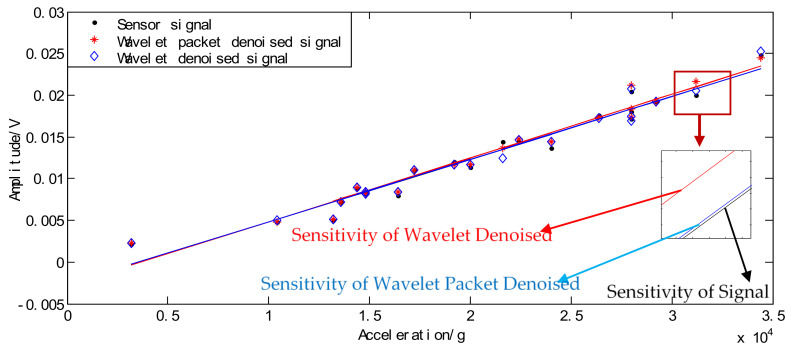
Static sensitivity fit diagram.

In order to compare the effects of two threshold denoising methods on the calibration of high-G accelerometers, the quasistatic calibration method was used to calibrate the sensitivity and spectrum of the sensor. The reference sensor was Endevco 2225A (as shown in Figure 14). The mean sensitivity was calculated by linear fitting the original signal and the denoised signal. The fitting curve is shown in Figure 15.

The original signal output curve obtained by calculation is: y = 7.4988 × 10^−7^x − 0.0026833; sensitivity is 0.74988 μv/g. The output curve after wavelet packet threshold denoising is: y = 7.6428 × 10^−7^x − 0.0028154; sensitivity is 0.76428 μv/g. The output curve after wavelet threshold denoising is: y = 7.5010 × 10^−7^x − 0.0026719; sensitivity is 0.7501 μv/g. After both denoising, the reconstructed signal increased the amplitude of the signal. However, the sensitivity and zero drift of the wavelet threshold denoising are closer to the original signal and more responsive to real sensor performance.

**Figure 16:**
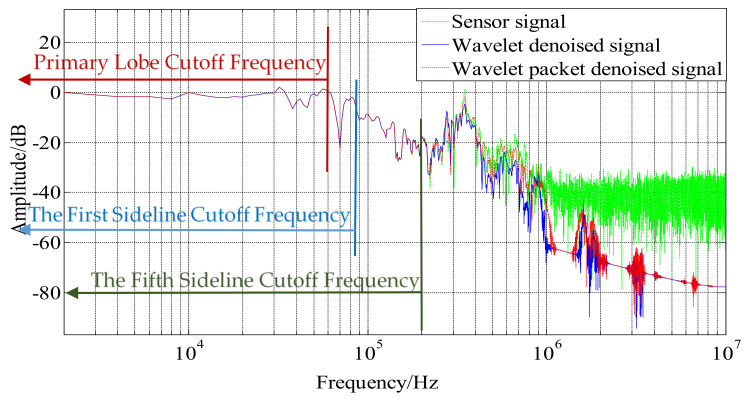
Normalized amplitude-frequency curve before and after denoising.

The amplitude–frequency curve (as shown in Figure 16) usually consists of a main lobe and several sidelobes, in which the lobe with the largest signal amplitude is called the main lobe and the rest are called sidelobes. According to the narrow pulse calibration theory mentioned by Zu Jing [27], the excitation pulse width required for narrow pulse calibration is mainly related to the frequency of the first main lobe, and the excitation signal normalizes the frequency of the intersection of the sidelobe envelope and the 0 dB line of the amplitude–frequency characteristic. It can be seen from the normalized amplitude spectrum before and after denoising in Figure 16 that the two denoising methods mainly affect the signal after the fifth side lobes. which are doped with a large number of non-excitation signals. Therefore, the two denoising methods have no effect on the calibration of the sensor. The research on sensor-related self-oscillation and package form has a great influence.

In order to compare the two denoising methods, Table 3 lists four denoising performance indicators: peak, integral value, signal-to-noise ratio (SNR), and root mean square error (RMSE). The peak value of the signal after the wavelet threshold denoising is increased by 3.4%, and the peak value of the wavelet packet threshold denoising is increased by 2%. The SNR is different from previous simulations, and the SNR of wavelet threshold denoising is reduced. The main reason is that the SNR of the simulation is based on the standard signal as the original signal, while the SNR of the test is based on the test signal with noise signal as the original signal. Therefore, the reduction of the SNR and the value of the integral value can explain that the wavelet threshold denoising ability is greater than the wavelet packet denoising ability and does not conflict with the previous simulation results. The RMSE of the two denoising methods is less than 0.2, and the RMSE of the wavelet packet threshold denoising method is slightly smaller than the wavelet threshold denoising method (one RMSE is 0.109, the other is 0.0916), which shows that the wavelet packet threshold denoising method in the high-frequency phase better retains the signal waveform, and can better reflect the high-frequency detail information.

## 5. Conclusions

In this paper, two denoising methods which are more suitable for HGMA are proposed: wavelet threshold denoising and wavelet packet threshold denoising. Stein’s unbiased likelihood estimation method was used as the threshold function, and the soft threshold was used to denoise the Machete hammer test signal. The wavelet threshold denoising method adopts adaptive layering, and the wavelet packet threshold denoising uses Shannon criterion for optimal tree searching. Compared with current single denoising methods, such as empirical mode decomposition (EMD) and local mean decomposition (LMD), the proposed method achieves a balance between denoising effect and signal fidelity. In the case of dynamic calibration, wavelet threshold denoising can increase the signal without changing the signal pulse width, which is more beneficial to the calibration of the sensor. Wavelet packet denoising has a higher SNR and is more suitable for studying parameters such as the influence of the sensor’s own packet on subsequent oscillation.

## Figures and Tables

**Table 1:** Resonant frequencies of the four modes.

Mode Shapes	1	2	3	4
**Resonant Frequency/kHz**	408	667	671	1119

**Table 2:** Denoising results of the two methods.

Denoising Method	SNR	RMSE
**Wavelet Threshold Denoising**	20.412	0.200
**Wavelet Packet Threshold Denoising**	12.875	0.151
**LPF Denoising**	17.829	0.270

**Table 3:** Summary of the denoising performance of the two methods.

Parameter	Sensor Signal	Wavelet Threshold Denoising	Wavelet Packet Threshold Denoising
**Peak (g)**	34,070	35,225	34,759
**Integral Value**	3.297 × 10^−4^	2.959 × 10^−4^	3.084 × 10^−4^
**SNR**		11.518	13.042
**RMSE**		0.109	0.0916

## Data Availability

Not applicable.
